# Supplementary material for: HFE Genotype, Ferritin Levels and Transferrin Saturation in Patients with Suspected Hereditary Hemochromatosis
Source: Genes (Basel). 2021 Jul 28;12(8):1162. doi: 10.3390/genes12081162 (PMC8394043; doi:10.3390/genes12081162)
Supplement: Supplementary file 1 [file genes-12-01162-s001.zip › genes-1284253-supplementary.pdf]

genes

1. Supplementary Data

Table S1. Genotypic differences in age and iron status.

**Genotypes**		**Age** Men	**Age** Women	**Ferritin** Men	**Ferritin** Women	**Transferrin saturation** Men	**Transferrin saturation** Women
C282Y/C282Y	C282Y/H63D	≤ 0.01	≤ 0.05	≤ 0.001	ns (0.475)	≤ 0.001	≤ 0.001
C282Y/C282Y	H63D/H63D	≤ 0.05	ns (0.236)	ns (0.163)	ns (0.807)	≤ 0.001	ns (0.051)
C282Y/C282Y	C282Y/wt	≤ 0.001	ns (0.352)	≤ 0.01	ns (0.636)	≤ 0.001	≤ 0.001
C282Y/C282Y	H63D/wt	≤ 0.001	≤ 0.05	≤ 0.05	ns (0.807)	≤ 0.001	≤ 0.001
C282Y/H63D	H63D/H63D	ns (0.953)	ns (>0.99)	ns (0.136)	ns (>0.99)	ns (0.195)	ns (0.348)
C282Y/H63D	C282Y/wt	ns (0.461)	ns (0.917)	ns (0.272)	ns (0.795)	≤ 0.001	ns (0.506)
C282Y/H63D	H63D/wt	ns (0.249)	ns (0.796)	≤ 0.05	ns (0.276)	≤ 0.001	ns (0.148)
H63D/H63D	C282Y/wt	ns (0.574)	ns (0.876)	ns (0.222)	ns (0.755)	ns (0.098)	ns (0.189)
H63D/H63D	H63D/wt	ns (0.271)	ns (>0.99)	ns (0.763)	ns (0.648)	≤ 0.01	ns (0.097)
C282Y/wt	H63D/wt	ns (0.642)	ns (0.974)	ns (0.160)	ns (0.636)	≤ 0.05	ns (0.883)

* Medians of parameters in respective genotypes have been pairwise compared using Mann-Whitney *U*-test

wt, wildtype; ns, not significant.

The table demonstrates genotypic differences in age and iron status expressed as *p*-values*.

Table S2. Correlation between ferritin, age, and biochemical parameters.

	**Age**				**Transferrin saturation**				**CRP**				**ALAT**				**GGT**			
	Men		Women		Men		Women		Men		Women		Men		Women		Men		Women	
Genotype	r	*p*-value	r	*p*-value	r	*p*-value	r	*p*-value	r	*p*-value	r	*p*-value	r	*p*-value	r	*p*-value	r	*p*-value	r	*p*-value
C282Y/C282Y	0.4503	≤ 0.001	0.3937	≤ 0.01	0.3064	≤ 0.01	0.1384	ns (0.370)	0.1817	ns (0.088)	0.4287	< 0.01	0.4219	≤ 0.001	0.2121	ns (0.166)	0.2405	≤ 0.05	0.1732	ns (0.261)
C282Y/H63D	0.2056	ns (0.095)	0.2597	ns (0.255)	0.0502	ns (0.686)	0.2432	ns (0.288)	0.3384	≤ 0.01	-0.2119	ns (0.369)	0.3549	≤ 0.01	0.5403	≤ 0.05	0.4180	≤ 0.001	0.1232	ns (0.594)
H63D/H63D	0.0961	ns (0.678)	-0.1000	ns (0.950)	0.162	ns (0.482)	0.8208	ns (0.133)	0.1112	ns (0.631)	0.2108	ns (0.833)	0.5429	≤ 0.05	0.9747	≤ 0.05	0.2793	ns (0.220)	0.9000	ns (0.083)
C282Y/wt	0.2005	ns (0.112)	0.3571	ns (0.444)	0.1097	ns (0.388)	0.6307	ns (0.141)	0.3986	≤ 0.01	-0.4364	ns (0.339)	0.2611	≤ 0.05	0.3571	ns (0.444)	0.3578	≤ 0.01	-0.6429	ns (0.138)
H63D/wt	0.0720	ns (0.790)	0.2256	ns (0.381)	0.3011	≤ 0.05	0.1475	ns (0.582)	0.0362	ns (0.769)	0.5448	≤ 0.05	0.1476	ns (0.226)	0.3996	ns (0.125)	0.7755	ns (0.526)	0.4503	ns (0.081)
Total	0.2193	≤ 0.001	0.3038	≤ 0.01	0.2409	≤ 0.001	0.1896	ns (0.068)	0.2052	≤ 0.001	0.2368	≤ 0.05	0.2906	≤ 0.001	0.3091	≤ 0.01	0.2061	≤ 0.001	0.1990	ns (0.055)

The table demonstrates correlation between ferritin and age, transferrin saturation, C-reactive protein (CRP), alanine aminotransferase (ALAT) and gamma-glutamyl transferase (GGT).
